# Iron Deficiency and Iron‐Deficiency Anemia Among Reproductive‐Age Women in the United States: Prevalence and Disparities, 2015–2023

**DOI:** 10.1155/anem/7951443

**Published:** 2026-08-27

**Authors:** Pranav Singh, Sabaa Saad-Omer, Carlos Galvez, Shweta Gupta

**Affiliations:** ^1^ Department of Internal Medicine, John H. Stroger Jr. Hospital of Cook County, Chicago 60612, Illinois, USA, cookcountyhhs.org; ^2^ Department of Hematology and Oncology, University of Illinois Chicago, Chicago 60612, Illinois, USA, uic.edu; ^3^ Division of Hematology-Oncology, John H. Stroger Jr. Hospital of Cook County, Chicago 60612, Illinois, USA, cookcountyhhs.org

**Keywords:** health equity, iron deficiency, iron-deficiency anemia, pregnancy, women’s health

## Abstract

**Introduction:**

Iron deficiency (ID) and iron‐deficiency anemia (IDA) remain highly prevalent among U.S. reproductive‐age women, with substantial racial, ethnic, and socioeconomic disparities. This study examined the contemporary burden of ID and IDA among U.S. reproductive‐age women using inflammation‐adjusted diagnostic criteria and an explicit health equity framework.

**Methods:**

Cross‐sectional analysis of 3322 women aged 18–49 years from the National Health and Nutrition Examination Survey (2015–2023). ID and IDA were defined using World Health Organization 2020 inflammation‐adjusted ferritin thresholds incorporating C‐reactive protein. Multivariable logistic regression examined associations between sociodemographic factors and iron status outcomes.

**Results:**

Among nonpregnant women (*n* = 3156), 27.4% had ID and 8% had IDA. Among pregnant women (*n* = 166), 75.5% had ID and 8.3% had IDA. Non‐Hispanic Black women had 2.8‐fold higher odds of IDA (adjusted odds ratio [aOR] 2.8, 95% CI 1.6, 4.9). Obesity was independently associated with ID (aOR 2.4, 95% CI 1.86, 3.24) in nonpregnant women and pregnant women (aOR 4.5, 95% CI 1.08, 18.76). Racial and ethnic disparities persisted after adjustment for education, income, and food security.

**Conclusions:**

ID and IDA remain highly prevalent among U.S. reproductive‐age women, with substantial and persistent racial, ethnic, and socioeconomic disparities. As a preventable and treatable condition with significant impacts on quality of life and pregnancy outcomes, it warrants greater clinical and public health attention, particularly among non‐Hispanic Black women, Hispanic women, women with obesity, and those experiencing socioeconomic disadvantages.

## 1. Introduction

Iron deficiency (ID), with or without anemia, continues to be one of the most prevalent and consequential nutritional deficiencies worldwide and affects millions of individuals in high‐income countries, including the United States [1–3]. Globally, from 1990 to 2021, the cases of iron‐deficiency anemia (IDA) have increased from 588 million to 826 million [4]. In high‐income countries, ID affects a significant portion of women of reproductive age, including approximately 38% with nonanemic ID and 13% with IDA, with prevalence increasing to over 80% during late pregnancy [1]. Based on a recent study in the United States using NHANES data from 2003 to 2023, overall prevalence of ID and IDA in pregnant women is 52.9% and 6.9%, respectively [5]. Among women of reproductive age, ID is particularly common due to a combination of increased physiological demands, menstrual blood loss, pregnancy‐related iron requirements, and dietary factors [1, 6]. Even in the absence of anemia, ID is associated with fatigue, impaired cognitive and physical performance, reduced quality of life, and adverse pregnancy outcomes, highlighting its clinical and public health relevance [1, 2, 7, 8].

Adolescence and early adulthood are critical periods during which depleted iron stores may never be adequately replenished, predisposing individuals to persistent deficiency throughout the reproductive years [9]. Despite this well‐established biological vulnerability, ID in reproductive‐age women often remains under‐recognized and undertreated, particularly when anemia is absent [1, 6]. Pregnancy represents a distinct physiological state that substantially amplifies iron requirements. Increased demands arise from expansion of maternal red cell mass, placental development, and fetal iron accretion, with requirements rising steeply across trimesters and during lactation [1, 10]. IDA in pregnancy is associated with a range of adverse maternal, fetal, and neonatal outcomes, including increased risk of postpartum depression and postpartum hemorrhage in mothers, and low birth weight, preterm birth, neonatal anemia, impaired cognitive and developmental outcomes, and increased risk of infections in fetuses and neonates [11, 12].

Racial, ethnic, and socioeconomic disparities compound these biological vulnerabilities. Prior studies demonstrate that ID and IDA disproportionately affect individuals facing structural and social disadvantages, including lower income, food insecurity, limited access to healthcare, and lack of insurance coverage [2, 13]. Racial and ethnic disparities are also well documented, with higher prevalence of IDA observed among Hispanic, Black, and other marginalized populations [13–15]. Accordingly, ID should not solely be understood as a nutritional or hematologic disorder, but as a condition shaped by social and structural inequities [2, 13].

Accurate estimation of ID prevalence is further complicated by ongoing challenges related to definition and measurement. Ferritin‐based thresholds for defining ID vary across studies, and ferritin levels are influenced by inflammation, potentially leading to underdiagnosis in populations with higher inflammatory burden [2, 16]. Recent work demonstrates that prevalence estimates and observed disparities can differ substantially depending on the diagnostic criteria used [17]. These definitional issues are not merely methodological. They have direct implications for clinical recognition, treatment decisions, and the equitable allocation of public health resources, particularly among populations with higher inflammatory burden who are most likely to be misclassified as iron‐replete under conventional unadjusted criteria. Ferritin behaves as an acute phase reactant, rising in the presence of inflammation independently of true iron stores [18]. As a result, populations with higher rates of chronic low‐grade inflammation, including women with obesity, women experiencing chronic psychosocial stress, and racial and ethnic minority groups, are at particular risk of having ID masked by spuriously elevated ferritin concentrations. Conventional ferritin thresholds developed without inflammation correction may therefore systematically underestimate ID prevalence and perpetuate diagnostic inequity in the populations already facing the greatest burden of disease. The WHO 2020 guidelines address this directly by raising the ferritin cutoff to less than 70 ng/mL in individuals with C‐reactive protein (CRP) of 5 mg/L or greater, enabling identification of ID that would otherwise go undetected [19]. Adopting these guidelines in population‐level surveillance is therefore not only methodological refinement but an equity imperative, ensuring that the most vulnerable populations are not rendered invisible by a diagnostic framework that does not account for their physiological context.

Although several nationally representative studies have evaluated ID and IDA in the U.S. population, important gaps remain. First, many prior analyses focus on children, adolescents, or pregnant individuals in isolation, while fewer studies center specifically on reproductive‐age women as a distinct equity‐relevant group warranting dedicated surveillance [9, 14]. Second, while recent analysis by Weyand et al. examined ID and IDA among pregnant individuals using NHANES data from 2003 to 2023, it was restricted to the pregnant cohort, did not apply inflammation‐adjusted ferritin criteria, and as a likely consequence, identified no significant sociodemographic associations with ID, suggesting that unadjusted ferritin misclassified a meaningful proportion of iron‐deficient women as iron‐replete [5]. Third, no nationally representative study has simultaneously applied WHO 2020 inflammation‐adjusted ferritin thresholds to both pregnant and nonpregnant reproductive‐age women within an explicit health equity framework. Prior U.S. analyses acknowledging this limitation have called for more rigorous inflammation correction in future surveillance studies. Taken together, these gaps leave clinicians, policymakers, and public health practitioners without a current, guideline‐aligned picture of ID burden in the U.S. reproductive‐age women.

To address these gaps, we conducted a nationally representative analysis of U.S. women of reproductive age using NHANES data (2015–2023). The primary objectives of this study were: (1) to estimate the contemporary prevalence of ID and IDA among nonpregnant and pregnant reproductive‐age women using WHO 2020 guideline‐aligned diagnostic criteria; (2) to characterize sociodemographic and structural disparities in iron status across racial, ethnic, and socioeconomic subgroups within an explicit health equity framework; and (3) to examine temporal trends in ID and IDA prevalence across the 2015–2023 NHANES cycles which span the COVID‐19 pandemic. Improved understanding of these disparities are essential to inform targeted screening strategies, clinical practice, and public health interventions aimed at reducing inequities in iron‐related health outcomes.

## 2. Methods

### 2.1. Study Design and Data Source

We conducted a pooled cross‐sectional analysis using data from the NHANES, a nationally representative survey of the noninstitutionalized civilian U.S. population conducted by the National Center for Health Statistics using a complex, multistage probability sampling design. We combined data from the 2015–2016, 2017–2018, and 2021–2023 NHANES cycles. The 2019–2020 cycle was not included because data collection was interrupted by the COVID‐19 pandemic. Although data were drawn from these three NHANES cycles spanning 2015–2023, each cycle is independently cross‐sectional, and participants are not followed across cycles.

NHANES data are publicly available and deidentified. Therefore, this analysis was considered exempt from institutional review board oversight.

### 2.2. Study Population

We restricted the analytic sample to women aged 18–49 years. We included participants who had valid measurements for hemoglobin, serum ferritin, and CRP. We excluded individuals with missing iron biomarker data or other causes of anemia. Consistent with NHANES protocols, pregnancy status was determined by self‐reporting and urine pregnancy testing.

### 2.3. ID and Anemia Definitions

We defined ID and IDA according to the WHO 2020 guideline on use of ferritin concentrations to assess iron status in individuals and populations, incorporating CRP as an indicator of inflammation [19]. Ferritin is an acute‐phase reactant that rises with inflammation, potentially masking true ID. Adjusting the ferritin threshold upward when CRP ≥ 5 mg/L corrects for this inflammatory elevation and prevents underdiagnosis in populations with higher inflammatory burden.

Among nonpregnant women, we defined ID by a serum ferritin < 15 ng/mL when CRP < 5 mg/L and < 70 ng/mL when CRP ≥ 5 mg/L. We defined anemia as hemoglobin < 12 g/dL [^20^]. We defined IDA as the coexistence of ID and anemia.

Among pregnant women, we defined ID as serum ferritin < 30 ng/mL when CRP < 5 mg/L, consistent with ACOG guidance [20], and < 70 ng/mL when CRP ≥ 5 mg/L, applying the WHO 2020 recommendations for individuals with inflammation. We defined anemia as hemoglobin < 11 g/dL and IDA as the presence of both conditions [21].

### 2.4. Sociodemographic and Clinical Covariates

We included the following sociodemographic variables: age (18–24, 25–34, and 35–49 years), race and ethnicity (non‐Hispanic White, non‐Hispanic Black, Hispanic, non‐Hispanic Asian, and other race, including multiracial), educational attainment (< high school, high school graduate, > high school), and poverty‐income ratio (PIR), defined as the ratio of total family income to the federal poverty threshold. We assessed food security status using the U.S. Department of Agriculture Household Food Security Survey Module. We defined obesity as body mass index ≥ 30 kg/m^2^. We selected covariates a priori based on prior literature and relevance to ID and health equity.

### 2.5. Statistical Analysis

All analyses accounted for the NHANES complex survey design, incorporating examination weights, strata, and primary sampling units, with cycle‐specific weights rescaled for pooling of three survey cycles. Weighted prevalence estimates were calculated overall and across sociodemographic subgroups. Associations between participant characteristics and ID outcomes were examined using survey‐weighted logistic regression, with covariates selected a priori including age, race/ethnicity, educational attainment, PIR, food security, and obesity. Analyses were performed in Stata (StataCorp, College Station, TX). Statistical significance was defined as *p* < 0.05 (two‐sided).

## 3. Results

### 3.1. Study Population

A total of 3322 reproductive‐aged women were included in the analysis. Of these, 3156 (95%) were nonpregnant and 166 (5%) were pregnant. Among these, 16.6% were aged 18–24 years, 41.2% were aged 25–34 years, and 42.2% were aged 35–49 years (Table 1—counts represented in this table are unweighted).

**TABLE 1:** Characteristics of the women in the analysis (unweighted counts and weighted percentages).

Characteristics	Participants (*n* = 3322)
Pregnancy status	
Nonpregnant	3156 (95%)
Pregnant	166 (5%)
Age (in years)	
18–24	551 (16.6%)
25–34	1368 (41.2%)
35–49	1403 (42.2%)
Race/ethnicity	
Mexican American	489 (14.7%)
Other Hispanic	397 (11.9%)
Non‐Hispanic White	1187 (35.7%)
Non‐Hispanic Black	676 (20.4%)
Non‐Hispanic Asian	396 (11.9%)
Other race, including multiracial	177 (5.4%)
Education	
< High school	444 (13.4%)
High school graduate	608 (18.3%)
> High school	2268 (68.3%)
PIR	
< 1	695 (20.9%)
1–1.99	772 (23.3%)
≥ 2	1855 (55.8%)
Food Security	
Secure	1538 (71.7%)
Insecure	607 (28.3%)
Obesity	
No	1943 (58.5%)
Yes	1379 (41.5%)

*Note:* Values represent unweighted counts and weighted percentages.

Abbreviation: PIR, poverty‐income ratio.

The racial and ethnic composition was 35.7% non‐Hispanic White, 20.4% non‐Hispanic Black, 14.7% Mexican American, 11.9% non‐Hispanic Asian, 11.9% Other Hispanic, and 5.4% multiracial/other race.

Most participants had education beyond high school (68.3%), and over half had a PIR ≥ 2 (55.8%). Food insecurity was reported by 28.3% of respondents. Obesity was present in 41.5% of the sample.

#### 3.1.1. Trends

**FIGURE 1:**
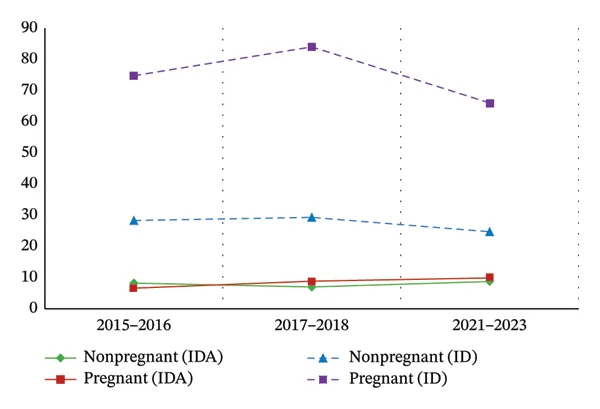
Trends in prevalence of ID and IDA across 2015–16, 2017–18, and 2021–23. IDA, iron‐deficiency anemia; ID, iron deficiency.

Figure 1 shows the trend in prevalence of ID and IDA across 2015–16, 2017–18, and 2021–23.

### 3.2. ID and IDA Among Nonpregnant Women

#### 3.2.1. Unadjusted Weighted Analysis

Among nonpregnant women, 27.4% had ID while 8% had IDA. ID prevalence varied substantially by race and ethnicity, with the highest rates among non‐Hispanic Black women (37.0%) and Mexican American women (36.7%), compared to non‐Hispanic White women (23.1%) and non‐Hispanic Asian women (22.5%). ID prevalence demonstrated significant socioeconomic gradients, with higher rates among women with lower educational attainment, lower income, food insecurity, and obesity. Age was not significantly associated with ID.

IDA showed even more pronounced racial and ethnic disparities, with prevalence of 19.0% among non‐Hispanic Black women, nearly five times higher than the 3.8% prevalence observed among non‐Hispanic White women. Mexican American and Other Hispanic women also experienced elevated prevalence (11.7%). IDA prevalence increased with age and decreased with higher educational attainment and higher income. IDA was also more prevalent among women with food insecurity and obesity (Table 2).

**TABLE 2:** Unadjusted sociodemographic analysis of ID and IDA in nonpregnant women.

Characteristics	Nonpregnant women					
	Iron deficiency			Iron‐deficiency anemia		
	Yes (%)	No (%)	*p* value	Yes (%)	No (%)	*p* value
*Age (in years)*						
18–24	26.8	73.2	0.75	6.7	93.3	0.055
25–34	26.7	73.3		7.2	92.8	
35–49	28.4	71.6		9.5	90.5	

*Race/ethnicity*						
Mexican American	36.7	63.3	< 0.001	11.7	88.3	< 0.001
Other Hispanic	31.7	68.3		11.7	88.3	
Non‐Hispanic White	23.1	76.9		3.8	96.2	
Non‐Hispanic Black	37	63		19	81	
Non‐Hispanic Asian	22.5	77.5		10.3	89.7	
Other race, including multiracial	28.8	71.2		5.9	94.1	

*Education*						
< High school	30.8	69.2	0.002	12.7	87.3	< 0.001
High school graduate	34.5	65.5		12.6	87.4	
> High school	25	75		6.1	93.9	

*PIR*						
< 1	32.2	67.8	0.02	10.9	89.1	0.018
1–1.99	29.5	70.5		8.7	91.3	
≥ 2	25.5	74.5		7	93	

*Food security*						
Secure	26.9	73.1	0.004	6.6	93.4	0.04
Insecure	34.6	65.4		9.8	90.2	

*Obesity*						
No	19.9	80.1	< 0.001	6.6	93.4	0.004
Yes	39.2	60.8		10.3	89.7	

Abbreviation: PIR: poverty‐income ratio.

### 3.3. Adjusted Weighted Analysis Among Nonpregnant Women

After adjusting for age, ethnicity/race, education, food security, and obesity, the following associations were observed (Table 3).

**TABLE 3:** Adjusted sociodemographic analysis of ID and IDA in nonpregnant women.

Characteristics	Nonpregnant women					
	Iron deficiency			Iron‐deficiency anemia		
	aOR	95% CI	*p* value	aOR	95% CI	*p* value
Age (in years)						
18–24	—	—	—	—	—	—
25–34	0.92	0.62, 1.37	0.69	2.3	1.19, 4.36	0.014
35–49	1.1	0.69, 1.63	0.76	3.2	2.04, 5.07	< 0.001
Race/ethnicity						
Mexican American	—	—	—	—	—	—
Other Hispanic	0.79	0.49, 1.29	0.34	1.32	0.66, 2.66	0.41
Non‐Hispanic White	0.59	0.42, 0.84	0.005	0.5	0.23, 1.1	0.08
Non‐Hispanic Black	1.08	0.77, 1.51	0.64	2.8	1.6, 4.9	0.001
Non‐Hispanic Asian	0.68	0.39, 1.17	0.15	1.7	0.74, 4.2	0.18
Other race, including multiracial	0.59	0.28, 1.25	0.16	0.35	0.12, 0.97	0.04
Education						
< High school	—	—	—	—	—	—
High school graduate	1.3	0.91, 1.97	0.12	1.54	0.9, 2.63	0.1
> High school	0.96	0.75, 1.23	0.78	0.61	0.38, 0.96	0.04
PIR						
< 1	—	—	—	—	—	—
1–1.99	1.1	0.77, 1.61	0.54	0.99	0.58, 1.67	0.97
≥ 2	0.97	0.66, 1.40	0.87	0.78	0.44, 1.4	0.4
Food security						
Secure	—	—	—	—	—	—
Insecure	1.1	0.85, 1.39	0.45	1.1	0.78, 1.51	0.58
Obesity						
No	—	—	—	—	—	—
Yes	2.4	1.86, 3.24	< 0.001	1.4	0.94, 2.11	0.09

*Note:* variables with “–” indicate the reference category.

Abbreviations: aOR, adjusted odds ratio; CI, confidence intervals; PIR, poverty‐income ratio.

Age was not significantly associated with ID. Compared with ages 18–24 years, IDA odds were higher in ages 25–34 and 35–49 years.

Non‐Hispanic Black women had 2.8‐fold higher odds of IDA, whereas non‐Hispanic White women had significantly lower odds of IDA, compared to Mexican American women. Women identifying as multiracial/other race had reduced odds of IDA.

Higher educational attainment was protective, with lower odds of IDA among women with education beyond high school. PIR was not significant in adjusted models. Obesity was independently associated with higher odds of ID, though not significantly associated with IDA after adjustment. Food security was not significant in fully adjusted models.

### 3.4. ID and IDA Among Pregnant Women

Given the small sample size of the pregnant subsample (*n* = 166), all analyses within this cohort should be considered exploratory and hypothesis‐generating and interpreted with appropriate caution.

### 3.5. Unadjusted Weighted Analysis

Among pregnant women, 75.5% had ID. The prevalence varied by race and ethnicity (*p* = 0.048), with the highest observed prevalence among non‐Hispanic White women (86.5%), followed by non‐Hispanic Asian women (72.9%) and Other Hispanic women (66.3%). Mexican American women (56.9%) and non‐Hispanic Black women (53.5%) had lower prevalence. Age and education were not significantly associated with ID (*p* = 0.44 and *p*=0.14, respectively).

IDA prevalence was 8.3% overall and demonstrated significant associations with educational attainment and income. The highest IDA prevalence was observed among women with less than high school education (21.9%) compared to those with greater than high school education (5.5%; *p* = 0.02). Income showed a nonlinear pattern, with the highest prevalence among women with PIR 1–1.99 (19.3%) compared to 12.7% among PIR < 1% and 3.9% among PIR ≥ 2 (*p* = 0.03). Race/ethnicity (*p* = 0.36), food security (*p* = 0.45), and obesity (*p* = 0.17) were not significantly associated with IDA. All subgroup findings should be interpreted cautiously given the limited pregnant sample size (*n* = 166).

### 3.6. Adjusted Weighted Analysis Among Pregnant Women

After adjusting for age, ethnicity/race, education, food security, and obesity, the following associations were observed (Supporting table 1).

Women aged 25–34 years had significantly lower odds of ID and IDA compared with women aged 18–24 years. High school graduates had significantly lower odds of IDA compared to women with less than high school education. Race and income were not significant predictors of ID or IDA after adjustment. Obesity was associated with significantly higher odds of ID. Food security showed no significant relationship in adjusted models.

## 4. Discussion

This nationally representative analysis reveals that ID and IDA remain highly prevalent among U.S. reproductive‐age women, with substantial racial, ethnic, and socioeconomic disparities. Using WHO 2020 inflammation‐adjusted criteria and recent NHANES data (2015–2023), we found that more than one in four nonpregnant women had ID, and one in 12 had IDA. Among pregnant women, the burden was markedly higher, with three in four experiencing ID. These findings confirm that ID continues to affect millions of women despite being preventable and treatable [1].

Temporal trends in ID and IDA prevalence were broadly stable across the three survey cycles. Among nonpregnant women, ID prevalence ranged from 28.3% in 2015–2016, 29.3% in 2017–2018, and 24.7% in 2021–2023, and IDA prevalence ranged narrowly from 7.0% to 8.7% (both nonsignificant). Among pregnant women, trends were more variable. ID prevalence was 74.8% in 2015–2016, rose to 84.0% in 2017–2018, and declined to 66.0% in 2021–2023. None of these differences reached statistical significance, likely reflecting the limited statistical power of the pregnant subsample (*n* = 166) to detect cycle‐to‐cycle differences. The COVID‐19 pandemic substantially disrupted healthcare utilization patterns, with documented declines in preventive care visits, prenatal care initiation, and routine screening [22, 23]. Although the 2019–2020 NHANES cycle was excluded from our analysis due to incomplete data collection, the 2021–2023 cycle captures a period of healthcare recovery that may not fully reflect prepandemic utilization patterns, particularly for populations with lower baseline access to care. Disruptions to prenatal care during this period are of particular relevance to our pregnant subsample, in whom routine iron screening and supplementation may have been delayed or inconsistently delivered. The modest, nonsignificant decline in ID prevalence in the most recent cycle may reflect postpandemic expansions in food assistance programs, including enhanced Supplemental Nutrition Assistance Program (SNAP) benefits [24]. Conversely, broader trends toward reduced dietary heme iron intake due to declining red meat consumption and increased reliance on ultraprocessed foods with lower iron bioavailability may have exerted countervailing pressure on iron status over this period [25, 26]. The overall stability of these estimates across nearly a decade underscores that ID has not meaningfully improved at the population level despite increased clinical and public health awareness [1, 2]. Taken together, these temporal factors introduce the possibility that our pooled prevalence estimates reflect a composite of distinct epidemiological conditions across cycles rather than a stable underlying prevalence, and cycle‐specific estimates should be interpreted with this heterogeneity in mind.

Persistent racial and ethnic disparities represent a critical equity concern. Non‐Hispanic Black women experienced nearly five times the rate of IDA compared to non‐Hispanic White women, with Mexican American and Other Hispanic women also disproportionately affected. These disparities persisted after adjustment for education, income, and food security, suggesting that biological, structural, and systemic factors beyond individual socioeconomic characteristics contribute to inequitable iron status [13–15]. Black women experience significantly higher incidence, prevalence, and severity of uterine fibroids compared to White women, leading to heavy menstrual bleeding and chronic iron loss [27]. Beyond the immediate clinical consequences, iron‐deficient mothers give birth to iron‐deficient neonates who face elevated risk of developmental deficits and enter reproductive age with depleted iron stores, compounding the population‐level burden across generations. Longitudinal evidence suggests that health consequences of racism are not self‐correcting but compound across generations [28].

Environmental factors further amplify biological vulnerability. Residential segregation and neighborhood‐level food insecurity limit access to quality food among Blacks, who are disproportionately concentrated in food‐insecure environments [29].

Structural factors in the healthcare system have also historically contributed to underdiagnosis of anemia in Black women. Until 2021, American College of Obstetrics and Gynecology guidelines applied race‐specific hemoglobin thresholds that set a lower diagnostic cutoff for anemia in Black women in the third trimester (hemoglobin < 10.2 g/dL for Black women versus hemoglobin < 11 g/dL for non‐Black women), effectively permitting a greater degree of anemia before triggering clinical action [30]. This threshold was subsequently abandoned after evidence demonstrated that Black women faced 65% increased risk of delivering with anemia compared to non‐Black women with hemoglobin in the same range [31]. The standard clinical definition of heavy menstrual bleeding requires patients to quantify blood loss using menstrual products, yet an estimated 16.9 million women in the United States live in poverty with inadequate access to menstrual products and hygiene facilities, effectively excluding the most economically vulnerable women from the accepted unit of measurement for this condition [32, 33]. The persistence of racial disparities in IDA in our study, even after adjustment for socioeconomic covariates, is consistent with this legacy of differential diagnostic standards and highlights the need for race‐conscious rather than race‐based approaches to ID screening and management.

Treatment access presents an additional and compounding layer of inequity. Oral iron supplementation remains the first‐line therapy for ID and IDA. However, when oral iron is poorly tolerated or ineffective, intravenous iron infusion is the preferred alternative [34, 35]. Intravenous iron is costly, and for individuals without adequate insurance coverage, out‐of‐pocket expenses may be prohibitive. Completing a standard course of two to five infusion appointments poses further logistical barriers for low‐income women, including cost of transportation, childcare, and work absenteeism. These structural barriers disproportionately affect the populations at the highest risk of IDA identified in our study and are likely to perpetuate the disparities we observe even when treatment is theoretically available.

The use of inflammation‐adjusted ferritin thresholds in this study is particularly important, as inflammation falsely elevates ferritin and can mask ID in populations with higher inflammatory burden [17]. Prior nationally representative estimates using conventional unadjusted thresholds highlight this concern. Using a ferritin cutoff of less than 15 ng/mL without inflammation adjustment across NHANES cycles from 1999 to 2023, Xiao et al. reported an overall ID prevalence of 17.3% and IDA prevalence of 6.2% among nonpregnant women aged 20–45 years. The most recent cycle (2021–2023) yielded estimates of 18.8% and 9.1% of ID and IDA, respectively, reflecting a significant upward trend over the study period [36]. Our estimate of 27.4% ID and 8.0% IDA, derived using WHO 2020 inflammation‐adjusted thresholds over a largely overlapping period, is substantially higher, a difference that is methodologically expected rather than discrepant. By raising the diagnostic ferritin threshold to less than 70 ng/mL when CRP is 5 mg/L or greater, our approach identifies iron‐deficient women whose ferritin is spuriously elevated by inflammation and who would be misclassified as iron‐replete under conventional criteria. The rising IDA trend documented by Xiao et al. independently corroborates our finding that the burden of ID in this population remains substantial and has not meaningfully improved at the population level despite increased clinical awareness [36].

Among pregnant women, the discrepancy between unadjusted and inflammation‐adjusted estimates is even more pronounced. Using a ferritin threshold of less than 30 ng/mL without inflammation adjustment across NHANES 2003 to 2023, Weyand et al. reported an ID prevalence of 52.9% in pregnant individuals [5]. Our estimate of 75.5%, derived using the same base ferritin cutoff but with WHO 2020 inflammation correction applied when CRP is 5 mg/L or greater, suggests that a substantial proportion of iron‐deficient pregnant women are missed when ferritin is interpreted without accounting for the physiological inflammatory state of pregnancy. Weyand et al. themselves acknowledged this as a limitation of their study, noting that prevalence estimates based on unadjusted ferritin may undercount affected individuals [5]. Our findings confirm this concern quantitatively and highlight the particular importance of inflammation‐adjusted diagnostic criteria in the pregnant population, where both iron demand and inflammatory burden are substantially elevated.

The clinical consequences of ID extend well beyond anemia and warrant explicit recognition. ID is associated with fatigue, impaired cognitive function, reduced physical performance, restless legs syndrome, and reduced quality of life [1, 7]. Recent evidence demonstrates that iron supplementation in individuals with nonanemic ID improves anxiety, fatigue, physical well‐being, and cognitive function [7]. Among reproductive‐age women, approximately 38% have nonanemic ID in high‐income countries, yet it frequently remains unrecognized because clinical focus centers on hemoglobin rather than iron stores [1, 37].

The pregnancy‐related findings are particularly concerning. ID during pregnancy is associated with preterm birth, low birth weight, preeclampsia, postpartum hemorrhage, and adverse neurodevelopmental outcomes in offspring [6, 10]. Beyond these obstetrical complications, maternal ID, even when corrected postnatally, is associated with persistent cognitive deficits and altered brain structure in children [38]. Our data suggest that more than three‐quarters of pregnant women meet criteria for ID using WHO inflammation‐adjusted thresholds, highlighting that ID remains common in pregnancy despite routine prenatal care. This burden exists against a backdrop of unresolved screening policy. While the U.S. Preventive Services Task Force recently concluded that evidence is insufficient to assess routine screening for ID and iron supplementation during pregnancy, professional societies, including the American College of Obstetricians and Gynecologists and the Centers for Disease Control and Prevention, recommend universal screening for anemia during pregnancy and low‐dose iron supplementation for at‐risk women [39]. Although iron supplementation effectively reduces ID and IDA at term, evidence linking treatment to improved clinical pregnancy outcomes remains limited [39, 40]. One observational study found that successful treatment response was associated with reduced odds of preterm birth (aOR 0.59) and preeclampsia (aOR 0.75), but this evidence has significant methodological limitations and does not establish causality [41].

Food insecurity emerged as a significant correlate of ID in unadjusted analyses, though this association was attenuated after adjustment for income and education. This pattern likely reflects complex inter‐relationships among poverty, food insecurity, and dietary quality. Food‐insecure households often have limited access to bioavailable heme iron from animal sources and may rely more heavily on plant‐based diets with lower iron bioavailability [9]. The attenuation suggests that income and education may partially account for the relationship between food insecurity and ID. Addressing ID in this population requires interventions that extend beyond supplementation, including expanding food assistance programs to improve access to nutrient‐dense foods [42, 43], Medicaid coverage for routine ID screening in reproductive‐age women, SNAP program enhancements targeting iron‐rich foods, advocating national guideline updates to address nonanemic ID, and targeted outreach for high‐risk groups identified in this study.

The strong independent association between obesity and ID has important mechanistic and clinical implications. Obesity‐related chronic inflammation increases hepcidin production, which inhibits intestinal iron absorption and sequesters iron in macrophages [44]. Notably, iron absorption in women with obesity is approximately two‐thirds that of normal‐weight women. The enhancing effect of ascorbic acid on iron absorption is also reduced by half [45]. This may contribute to persistent ID in women with obesity and reduced response to standard oral supplementation. Additionally, our study found a strong association between obesity and ID among pregnant women. This finding is clinically relevant in the context of prior studies demonstrating that infants born to mothers with obesity are at increased risk of ID, a condition associated with adverse neurodevelopmental outcomes [46–48]. These mechanisms warrant further prospective investigation.

This study has important strengths. The use of recent nationally representative NHANES data spanning 2015 to 2023 ensures that findings reflect contemporary burden of ID and IDA in the United States. The application of WHO 2020 inflammation‐adjusted diagnostic criteria represents a methodological advance over prior analyses using conventional unadjusted criteria, enabling more accurate estimation of true burden of ID and IDA. The simultaneous examination of both ID and IDA as distinct outcomes, rather than anemia alone, provides a more complete picture of the ID spectrum and its clinical relevance across the reproductive lifespan in women.

Several limitations should be acknowledged. The pooled NHANES cycles mean that participants are not followed across cycles. Structural changes in food access and healthcare utilization across this period may affect iron status estimates. The pregnant subsample (*n* = 166) was insufficient for stable stratified estimates. Adjusted findings for pregnant women should be considered hypothesis‐generating only. We were unable to assess trimester‐related data due to lack of standardized NHANES measurement. Additionally, forthcoming ASH guidelines are anticipated to recommend a uniform ferritin threshold of less than 30 ng/mL for both menstruating and pregnant individuals; future studies applying these guidelines alongside inflammation adjustment will be needed to produce fully comparable national estimates.

Several research gaps warrant attention. The optimal ferritin threshold for defining clinically meaningful ID remains contested, and head‐to‐head outcome comparisons across the threshold definitions are needed to establish evidence‐based cutoffs. Implementation research is needed to identify equitable screening delivery models that integrate ferritin testing into routine well‐woman and prenatal care, with particular attention to populations facing structural barriers to access. Future studies should assess whether weight management or anti‐inflammatory interventions improve iron status in women with obesity. Adequately powered trials are needed to determine whether correcting ID in pregnancy translates to meaningful reductions in maternal and neonatal morbidity.

## 5. Conclusion

The high prevalence of nonanemic ID defined in this study, affecting more than one in four nonpregnant reproductive‐age women and over three‐quarters of pregnant women, has important clinical and public health implications. Current screening guidelines focused on hemoglobin alone will miss the majority of iron‐deficient women, as our data confirm that most ID in this population occurs in the absence of anemia. These findings highlight the need for harmonized national screening guidelines that incorporate inflammation‐adjusted ferritin measurement as a routine component of well‐woman and prenatal care. Persistent racial and ethnic disparities in IDA observed in this and prior studies, compounded by historical use of race‐specific hemoglobin thresholds that systematically delayed anemia diagnosis in Black women, highlight the need to move toward race‐conscious rather than race‐based diagnostic frameworks, and to prioritize screening and treatment access for populations that bear the greatest burden of ID yet face the greatest structural barriers to its identification and management.

## Funding

The authors received no external financial support for the research, authorship, or publication of this article.

## Conflicts of Interest

The authors declare no conflicts of interest.

## Supporting Information

Additional supporting information can be found online in the Supporting Information section.

## Supporting information


**Supporting Information 1** Supporting table 1: Adjusted sociodemographic analysis of ID and IDA in pregnant women.

**Supporting Information 2** Supporting table 2: Data used to construct Figure 1. All authors have read and approved the final version of the manuscript. Pranav Singh had full access to all of the data in this study and takes complete responsibility for the integrity of the data and the accuracy of the data analysis.

## Data Availability

The data used in this study are publicly available from the National Center for Health Statistics. NHANES data can be accessed at https://www.cdc.gov/nchs/nhanes/
