# Supplementary material for: Iron Deficiency and Iron‐Deficiency Anemia Among Reproductive‐Age Women in the United States: Prevalence and Disparities, 2015–2023
Source: Anemia. 2026 Aug 27;2026:7951443. doi: 10.1155/anem/7951443 (PMC13519950; doi:10.1155/anem/7951443)
Supplement: Supplementary file 1 [file ANEM-2026-7951443-s001.docx]

**Supplementary data**

**Supplementary table 1:** Adjusted sociodemographic analysis of ID and IDA in pregnant women.

| **Characteristics** | **Pregnant women** | | | | | |
| --- | --- | --- | --- | --- | --- | --- |
|  | **Iron deficiency** | | | **Iron deficiency anemia** | | |
|  | **aOR** | **95% CI** | **p-value** | **aOR** | **95% CI** | **p-value** |
| **Age (in years)** |  |  |  |  |  |  |
| 18–24 | - | - | - | - | - | - |
| 25–34 | 0.03 | 0.001, 0.90 | 0.044 | 0.19 | 0.04, 0.89 | 0.035 |
| 35–49 | 0.02 | 0.002, 0.25 | 0.003 | 0.17 | 0.03, 1.09 | 0.06 |
| **Race/Ethnicity** |  |  |  |  |  |  |
| Mexican American | - | - | - | - | - | - |
| Other Hispanic | 1.2 | 0.15, 9.19 | 0.85 | 3.54 | 0.26, 48.9 | 0.33 |
| Non-Hispanic White | 5.14 | 0.65, 40.7 | 0.116 | 0.33 | 0.02, 4.56 | 0.39 |
| Non-Hispanic Black | 1.23 | 0.15, 10.17 | 0.84 | 1.23 | 0.09, 15.4 | 0.86 |
| Non-Hispanic Asian | 5.85 | 0.58, 59.11 | 0.13 | 2.90 | 0.21, 38.7 | 0.41 |
| Other Race- including multiracial | 0.95 | 0.04, 24.1 | 0.97 | 1.51 | 0.09, 23.2 | 0.31 |
| **Education** |  |  |  |  |  |  |
| < High school | - | - | - | - | - | - |
| High school graduate | 1.21 | 0.16, 9.17 | 0.84 | 0.13 | 0.03, 0.64 | 0.013 |
| > High school | 3.53 | 0.57, 21.8 | 0.166 | 0.3 | 0.06, 1.52 | 0.144 |
| **PIR** |  |  |  |  |  |  |
| <1 | - | - | - | - | - | - |
| 1-1.99 | 0.63 | 0.14, 2.84 | 0.55 | 1.92 | 0.22, 16.89 | 0.54 |
| ≥2 | 1.13 | 0.17, 7.28 | 0.44 | 0.89 | 0.034, 4.69 | 0.45 |
| **Food Security** |  |  |  |  |  |  |
| Secure | - | - | - | - | - | - |
| Insecure | 1.14 | 0.27, 4.89 | 0.85 | 1.2 | 0.25, 5.93 | 0.25 |
| **Obesity** |  |  |  |  |  |  |
| No | - | - | - | - | - | - |
| Yes | 4.5 | 1.08, 18.76 | 0.04 | 0.79 | 0.11, 5.95 | 0.81 |

aOR: Adjusted odds ratio, CI: Confidence intervals, PIR: Poverty-income ratio, variables with “–” indicate the reference category.

**Supplementary table 2:** Data used to construct Figure 1.

|  | **Iron deficiency anemia (IDA)** | | | | **Iron deficiency (ID)** | | | |
| --- | --- | --- | --- | --- | --- | --- | --- | --- |
| **Years** | Nonpregnant | p-value | Pregnant | p-value | Nonpregnant | p-value | Pregnant | p-value |
| 2015–2016 | 8.25 | 0.49 | 6.63 | 0.76 | 28.3 | 0.1 | 74.8 | 0.25 |
| 2017–2018 | 7 |  | 8.83 |  | 29.3 |  | 84 |  |
| 2021–2023 | 8.75 |  | 9.91 |  | 24.7 |  | 66 |  |
